# Neoangiogenesis in Melanoma: An Issue in Biology and Systemic Treatment

**DOI:** 10.3389/fimmu.2020.584903

**Published:** 2020-10-29

**Authors:** Davide Quaresmini, Michele Guida

**Affiliations:** Rare Tumors and Melanoma Unit, IRCCS Istituto Tumori “Giovanni Paolo II”, Bari, Italy

**Keywords:** ****neoangiogenesis, melanoma, combination strategy, antiangiogenics, immunotherapy

## Abstract

Neoangiogenesis is a recognized hallmark of cancer, granting tumor cells to dispose of metabolic substrates through a newly created vascular supply. Neoangiogenesis was also confirmed in melanoma, where vascular proliferation is associated with increased aggressiveness and poorer prognosis. Furthermore, melanoma cells show the so-called vascular mimicry, consisting in the assumption of endothelial-like features inducing the expression of pro-angiogenic receptors and ligands, which take part in the interplay with extracellular matrix (ECM) components and are potentiated by the ECM remodeling and the barrier molecule junction alterations that characterize the metastatic phase. Although neoangiogenesis was biologically proven and clinically associated with worse outcomes in melanoma patients, in the past anti-angiogenic therapies were employed with poor improvement of the already unsatisfactory results associated with chemotherapic agents. Among the novel therapies of melanoma, immunotherapy has led to previously unexpected outcomes of treatment, yet there is a still strong need for potentiating the results, possibly by new regimens of combination therapies. Molecular models in many cancer types showed mutual influences between immune responses and vascular normalization. Recently, clinical trials are investigating the efficacy of the association between anti-angiogenetic agents and immune-checkpoint inhibitors to treat advanced stage melanoma. This paper reviews the biological bases of angiogenesis in melanoma and summarizes the currently available clinical data on the use of anti-angiogenetic compounds in melanoma.

## Introduction

Melanoma is an aggressive cancer arising from melanocytic precursors, with high potential for locoregional and metastatic spread. As a common hallmark of cancers, when the dimensions of the primary tumoral mass of melanoma reach the threshold for nutrient diffusion, a cancer-specific net of blood vessels is fundamental to provide substrates for cancer cell survival and growth (1). The newborn neoplastic vasculature is aberrant and incomplete with distorted, dilated, and leaky vessels, insufficient pericyte coverage, abnormal endothelial cell proliferation, an uneven distribution within cancer tissues, and wide fenestrations that ultimately contribute to tumor cell sprouting through the vascular flow (2–5). The process of activation of quiescent vasculature by cancer cells is called “angiogenic switch” and is sustained by a disequilibrium between molecular activators and inhibitors of angiogenesis, in favor of angiogenesis (6). Angiogenesis is essential in growth and progression of cancers, including melanoma (7). Warren first described angiogenesis in a melanoma graft animal model, observing remarkable angiogenic capacity of melanoma tissue (8). Since then, angiogenesis in melanoma has been furtherly investigated to identify a possibly more complex and heterogeneous behavior in the handling of blood vessels by this neoplasm (9). First, melanoma cells induce the spread of new blood vessels by pre-existing ones; secondly, melanoma cells recruit bone marrow progenitors that reach hypoxic areas within the tumor microenvironment, where they can induce vascular formation; finally, melanoma cells themselves can acquire an endothelial-like phenotype in the so-called vascular mimicry phenomenon, directly taking part into the structure of blood vessels (9). So far, inhibitors of angiogenesis have been used in oncology in other cancers including renal, colorectal, and ovarian neoplasms, but data from clinical melanoma research are lacking. The current therapies for advanced stage disease in melanoma are based on the inhibition of either the aberrantly activated BRAF/MEK pathway, or the immune checkpoints PD1 and CTLA4. Despite obtaining previously unexpected outcomes in patients with advanced disease, leading to improved survival rates, these therapies still have potential for further improvements. This paper summarizes the biological bases of angiogenesis in melanoma matching to the most recent development of treatments, and reviews the currently available preclinical and clinical data on the use of anti-angiogenetic compounds in patients with melanoma.

## Insights Concerning the Molecular Mechanisms of Angiogenesis

The recent evolution of melanoma treatment has led to a primary role of targeted and immune therapy both in the adjuvant and the metastatic setting. Interestingly, the molecular architecture of melanoma microenvironment is far more complex than previously thought, and a strong cross-talk has been clearly demonstrated between the angiogenic and the immune components of cancer stroma, with mutual influences in the molecular compartments that are involved in response to systemic treatments. Angiogenic factors are produced by tumor cells, stromal cells including cancer-associated fibroblasts (CAFs), and inflammatory cells like lymphocytes and macrophages. The hyperproduction of pro-angiogenetic factors is induced by several mechanisms, primarily the activation of the hypoxia-induced HIF1α pathway, the oncogene-induced transcription of Vascular Endothelial Growth Factor (VEGF), and the loss of oncosuppressor genes including p53, which both stimulates the production of antiangiogenic factors like thrombospondin-1 and inhibits the expression of proangiogenic factors (10, 11).

### Vascular Endothelial Growth Factor (VEGF)

VEGF-A is the archetype and most biologically relevant among proangiogenic factors, with a strong effect on endothelial survival and migration (12), and on vasculogenic mimicking properties in melanoma cells (13). VEGF-A is upregulated by HIF-1α and oncogene signaling pathways (14–16). Remarkably, VEGF-A has also shown immunosuppressive capacity. In particular, the increased production of VEGF-A in cancer leads to inhibition of T cells in several ways: 1) reducing the activity of functional T cells both directly (17) and indirectly through the endothelial PGE2-mediated suppressive action on T cells (18); 2) decreasing neoantigen presentation to lymphocytes by inhibiting the maturation of dendritic cells (DCs) (19), mainly interfering with NF-kB activation (20); 3) recruiting immunosuppressive T regulatory cells (Tregs) into the tumor microenvironment (21, 22); 4) limiting endothelial cytokine response and adhesion molecule expression, hence affecting vascular functional permeability to leukocytes and their peripheral recruitment to cancer microenvironment (23, 24). Relevantly for immunotherapy, VEGF pathway activation also enhances T cell exhaustion mediated by immune checkpoints like PDL1, CTLA4, TIM3, and LAG3 (25). While lowering the immunogenic compartment of immunity, VEGF potentiates the counteracting immunopermissive microenvironment (26) both by recruiting immune-suppressive Tregs (27) and myeloid derived stromal cells (MDSCs) (28), and activating tumor-associated macrophages (TAMs) at the tumor site (29). To confirm this role of VEGF, the anti-VEGF antibody Bevacizumab induces DC maturation and a reduction in Tregs and MDSCs recruitment to cancer sites (22, 30, 31). VEGF can be produced by cancer cells and immune cells from tumor microenvironment, mostly from Tregs and in smaller proportions from TAMs, MDSCs and DCs (32), creating cellular communications that either directly or indirectly convey on the inhibition of cytotoxic T lymphocytes (33).

### Angiopoietin-2 (ANG-2)

ANG-2 is an antagonist cytokine of the Angiopoietin-1/Tie2 pathway that acts as a facilitator of VEGF-dependent angiogenesis (34). ANG-2 has long been considered an exclusive product of endothelium, but more recently Pari and colleagues demonstrated that it can also be produced by melanoma (35). ANG-2 levels in sera are increased in stage III and IV melanoma patients, but not in stages I and II (36). Consistently with this evidence, ANG-2 is produced by melanoma cells themselves, especially by metastatic sites (35). Differently from the stromal-derived ANG-2, melanoma-derived ANG-2 was not shown to increase the microvessel density of melanoma microenvironment but rather showed a protection of tumor cells from oxidative stress and a role in reactive oxygen species associated metastatization to the lungs in a mouse model (35). High serum ANG-2 levels were correlated with worse overall response rate to immunotherapy in melanoma (37). ANG-2 contributes to immune microenvironment composition, by acting on the Tie-2 expressing subpopulation of circulating monocytes, that are recruited by ANG-2 and converted to M2-like macrophages (38) and secrete IL10, which is a known promoter of Treg expansion and inhibitor of effector T cell activity (39).

### Toll-Like Receptors (TLRs)

TLRs are a family of pattern recognition receptors involved in antimicrobial immunity, apoptotic cell clearance, and cancer. Among all family members, TLR-4 is expressed in 90% of primary and 93% of metastatic melanomas, where it plays a role in the aggressive behavior of cancer cells (40). TLR-4 signaling involves the activation of signal transducer and activator of transcription 3 (STAT3), which on turn promotes melanoma growth and aggressiveness associated features including angiogenesis and epithelial to mesenchymal transition (41). During melanomagenesis, ultraviolet radiation recruits and activates neutrophils in a TLR4-mediated mechanism, inducing an inflammation that facilitates angiogenesis and favors melanoma angiotropism (40). Moreover, STAT3 has also been associated to immunosuppression in melanoma (42).

## Immune Cells in Microenvironment and Angiogenesis

In cancer microenvironment, there is a constant dynamic cross-talk between all resident cells which is far beyond the mere activity of cancer cells alone: the dynamic interaction between all cell components is responsible for the biological behavior of cancer. Accordingly, angiogenesis in cancer is not only induced by cancer cells themselves: the immune cells in tumor microenvironment can sustain angiogenesis in cancer (43). Globally, tumor cells can influence immune infiltrates towards an immune permissive phenotype. VEGF, for example, is produced by TAMs, tumor-associated neutrophils, regulatory DCs, myeloid derived suppressor cells, NK cells, and γδT17 cells (43). VEGF-R1 and -2 are expressed on DCs, which can promote angiogenesis (44). Neutrophils and TAMs secrete proangiogenic factors including VEGF, TNFα, IL8, and chemokines (45), together with matrix metalloproteases, which are essential to remodel the extracellular matrix during angiogenesis and metastatization (46). In an analysis on mouse models of breast cancers, extended to some of TCGA databases excluding melanoma, Tian et al. (47) demonstrated that the activation of vessel normalization (pericyte coverage, reduced vascular leakage, improved blood perfusion) is one of the effects of immune checkpoint inhibitors (ICIs) mediated by the activation of CD4+ T lymphocytes, in particular IFNγ producing Th1 cells (47). In particular, the authors postulated the existence of a positive feedback loop, according to which Th1 cells localize proximal to tumoral vessels and change the local tumor microenvironment *via* CKs like IFNγ, which induces a reduction in VEGF-A production and an increase in Th1- and pericyte-recruiting chemokines CXCL9, CXCL10, CXCL11 (47). Such finding would confirm the evidence that inhibition of IFNγ signaling is associated to secondary resistance to immune checkpoint inhibitors (48). Furthermore, after interacting with DCs, T cells can acquire neurophilin-1 which is a ligand of VEGF-A that promotes angiogenesis (49). Endothelial cells, formerly considered passive lining cells of blood vessels, are actively responsible of the intense reciprocal cellular interaction consisting in the so-called angiocrine signaling that is essential in normal organ development (50) and can be exacerbated in cancer. Tumor cells can then induce endothelial activation mediated by CKs including Angiopoietin-2, which are responsible for the autocrine induction of STAT3 signaling in the endothelium, followed by the expression of chemokines (CCL2) and adhesion molecules (ICAM1) that recruit CCR2+ macrophages to the cancer site (37). Endothelial cells take part in granulocyte differentiations in physiology and pathology, given the common developmental origin between endothelial cells and hematopoietic cells (37): the endothelium secretes CKs (SCF, CXCL12) that contribute to the quiescence of hematopoietic cells in the bone marrow, but can also promote granulopoiesis in case of inflammation (51). The hypoxia-regulated Endothelin B receptor on tumor endothelium acts as an obstacle to T cell adhesion and has been identified in some cases of resistance to immune therapy (52).

## Manipulation of Angiogenesis by Therapeutic Agents in Melanoma

Antiangiogenics are a class of kinase inhibitors that bind either angiogenic factors or their receptors. The first antiangiogenic agent to be developed was Bevacizumab, an anti-VEGF monoclonal antibody, which is still indicated in the treatment of cancers including colorectal, ovarian, or uterine carcinomas. Many other agents were synthesized furtherly, presenting a wider spectrum of pharmacodynamic targeting, including Sunitinib, Pazopanib, Ramucirumab, Regorafenib, Sorafenib, Aflibercept, being so far approved in daily practice either alone or in combination. Antiangiogenic agents model the irregular and leaky vessels of cancer to create an almost normalized intratumoral vascular network, at least transiently. Such improved vascular efficiency of cancer microcirculation is thus the main responsible for a more efficient transport of chemotherapic agents to cancer cells, and also the molecular background to the association of antiangiogenic therapies with traditional chemotherapies (53). Part of the effects of antiangiogenics in cancer may also be attributed to the modulation of immune cell composition in tumor microenvironment, triggered by the reduction in the tissue hypoxia that is associated with the immature cancer vasculature. The response to hypoxia favors the polarization of the tumor microenvironment towards an immune-suppressive phenotype in terms of increase in Tregs and M2-TAMs, reduction of DC activity, and increase in PDL1 expression on endothelial cells, TAMs, DCs, and cytotoxic lymphocytes (33). Antiangiogenics can then interfere with both CD8+ T cells trafficking and TAMs repolarization, inducing an immunostimulatory milieu (37). Anti-VEGF-A agents also improve immune responses (54). In a preclinical model of antiangiogenic-driven vascular normalization in melanoma and other primary cancers, Schmittnaegel et al. demonstrated that the administration of a bispecific anti-VEGF-ANG2 antibody was associated with increased recruitment and activation of CD4+ and CD8+ T cells at the areas with normalized vessels (55). In the cross-talk between adaptive and innate immunity, IFNγ from CD4+ and CD8+ T cells then stimulates M1-TAMs to angiostatic activity and antagonize endothelial cell proliferation (56). In a preclinic mouse model with multiple cancer cell lines including melanoma, DeAlmeida and Colleagues demonstrated that therapy with anti-VEGFA targeting agent induces the HIF1α-mediated activation of intratumoral CD8+ cells resulting in an increase in IFNγ and TNFα production (57). Unfortunately, the response is usually transient (58) and short in duration (59), due to mechanisms of escape that include the activation of metabolic stress responses in cancer cells; the activation of alternative angiogenic pathways like the ANG-2 pathway; the participation to new vessel growth according to the vascular co-option mechanism; the normalization of cancer vessels (56). In renal cell carcinoma, two different molecular subsets have been identified: the angiogenic subset, characterized by upregulation of angiogenesis-associated genes and responsiveness to antiangiogenic therapy, and the inflammatory subset, presenting an upregulation in immune-related genes and refractoriness to antiangiogenic therapy (60). In contrast, no similar dichotomy has been identified in melanoma, for which resistance to antiangiogenic therapies may be intrinsically associated to its biology: as evidenced by Donnem, melanoma primary and metastatic lesions strongly rely on vessel co-option for their vascular supply, as an alternative to angiogenesis (61). In melanoma, the mutation of the BRAF gene has primary importance, since mutations at the codon 600 are druggable and clinically associated with significant responses. The constitutive activation of BRAF kinase is not only a driver mutation in melanoma, but also has effects on melanoma microenvironment composition (62). In particular, BRAF mutations are associated with an increased density of Treg infiltrate (63), and a reduction in T cell activity by the indirect secretion of IL-1α and IL-1β from CAFs (64). PDL1 expression in immune infiltrate is also more prevalent in BRAF mutated tumor specimens (65). Finally, BRAF mutated cells also have angiogenic capacity (66), since they can induce other cancer cells and microenvironmental cells to secrete CXCL8 and CCL2, two pro-tumorigenic chemokines, leading to cancer cell proliferation and macrophage-mediated angiogenesis (62). Furthermore, the pathway of VEGFR-1 has recently been identified as an escape mechanism to the BRAF-inhibitor Vemurafenib (67). As evidenced by Aztori and colleagues, melanoma cell lines express higher VEGF-receptors when transformed into Vemurafenib-resistant, and silencing of such receptors can prolong the maintenance of sensitivity to Vemurafenib (67). Together with targeted therapy for BRAF mutated patients, the backbone of treatment in advanced melanoma is represented by the immune therapy with either anti-CTLA4 or anti-PD1 agents, that remove immune checkpoint inhibition to potentiate the immune response to melanoma. Despite an overall improvement in the outcomes of treatment, still immune therapy is often associated to secondary resistance and progression and, yet more rarely, to early resistance. Among the possible mechanisms underpinning these resistances, recent evidence also identified some vascular-related mechanisms. In particular, high serum ANG-2 levels correlated with worse overall response rate to ICI therapy in melanoma, possibly because ANG-2 can recruit monocytes and induce PDL1 expression in M2-macrophages (37). Wu and colleagues identified a subset of melanoma patients characterized by a significant tumor infiltration of CD68+ macrophages that particularly responded to treatment with Ipilimumab and Bevacizumab with a neat decrease in ANG-2 expression (68). Allen and colleagues demonstrated that the combination therapy of antiangiogenics and anti-PD1 agents induced an increase in intratumoral high endothelial venules responsible for selective leukocyte infiltration and for the switch of microenvironment towards immunosensitive features (69). A bispecific anti-VEGFA and anti-ANG2 was also shown to potentiate the efficacy of an anti-PD1 treatment (55).

## Discussion: Evidence From Clinical Treatment Outcomes and Possible Perspectives

The first trials involving antiangiogenetic drugs in melanoma date back to early experiences two decades ago, when chemotherapy was the only available treatment for advanced stage disease, with palliative intent and dramatically poor outcomes. Most of these studies are phase 1 or 2 trials for stage III unresectable or stage IV melanoma, either in single or double arm of treatment, with small cohorts of recruited patients, usually 20–30 (**Table 1**). Despite preclinical data suggesting the advantage of a more regular vascular network in the distribution of chemotherapics to cancer cells, these trials did not provide satisfying results from the association of antiangiogenics with common chemotherapics, showing no statistically significant improvement in the outcomes of traditional chemotherapy schedules, therefore they have never been investigated in wider phase 3 clinical trials and have never been adopted in everyday practice. More recently, immune therapy became the new gold standard for systemic therapy, together with anti-BRAF and anti-MEK targeted therapy for BRAF mutated patients, and was tested in the association with antiangiogenic agents, given the evidence of efficacy from the combination of antiangiogenics with ICIs not only in preclinical, but also in the clinical settings for other cancers including renal clear cell or non-small cell lung cancer. Hodi and colleagues performed an investigational phase I trial in 46 patients with advanced melanoma without brain metastases receiving a first (37%) or second (63%) line treatment with Ipilimumab and Bevacizumab: the best overall response rate (ORR) was 19.6% with a disease control rate of 67.4% and a median time to progression of 9 months (78). The immunohistochemical analysis of serial biopsies of target lesions revealed changes in melanoma-associated endothelium with increased expression of E-selectin (78). In a more recent phase IB/II trial, Taylor and colleagues treated patients with advanced solid tumors including melanoma with the association of Pembrolizumab and Lenvatinib, an inhibitor of multiple kinases including VEGFR. Among the 21 patients of the melanoma subcohort, the ORR was 33% and also included 1 complete response, while the disease control rate was 81% (79). Recently, Arance and colleagues presented the preliminary results of the phase 2 LEAP004 trial investigating the association of Lenvatinib and Pembrolizumab in 103 patients with advanced melanoma progressing on immunotherapy in second or further line of treatment. The median progression-free survival was 4.2 months with a median overall survival of 13.9 months and a 21.4% response rate, supporting a possible role in overcoming resistance to immunotherapy by Lenvatinib (80). These encouraging data are now furtherly being investigated in a randomized phase III trial specifically dedicated to advanced melanoma. Moreover, other studies are currently ongoing, mostly still in the recruitment phase, for treatment associations of antiangiogenics with anti-PD1 (Nivolumab or Pembrolizumab) or anti-PDL1 (Avelumab) inhibitors (**Table 2**). As previously described, angiogenesis plays a major role in the natural history of melanoma, from its intrinsic aggressiveness to some forms of resistance to systemic therapy. Despite widely intertwined mechanisms between angiogenesis and immunity, the efficacy of antiangiogenic therapies is currently insufficient. Hence, much interest is addressed to the ongoing clinical trials of combined antiangiogenic and immune therapies, to pursue better outcomes in the therapy of advanced melanoma.

**Table 1:** Clinical trials of the association of antiangiogenic treatments with old drugs in advanced melanoma.

Reference	Phase	Clinical setting	Line of treatment in metastatic setting	Arm 1	Arm 2	Primary endpoints	Secondary endpoints	Further analyses
Del Vecchio et al. (70)	II	Stage IV cutaneous melanoma.	1^st^ line	Bevacizumab + Fotemustine (20 pts)	None	CR 1/20 PR 2/20 SD 10/20	TTP 8 m OS 20 m G3 toxicity 14/20 pts	Reduction of VEGF levels post-therapy
Tarhini et al. (71)	II	Stage III unresectable or stage IV cutaneous melanoma. No active brain metastases.	1^st^ line or further	Aflibercept (40 pts)	None	ORR 7.5%	PFS 4 m Os 16 m	Hypertension correlated with OS
Von Moos et al. (72)	II	Stage IV cutaneous melanoma. No brain metastases.	1^st^ line	Bevacizumab + Temozolomide (62 pts)	None	SD 52%	ORR 16% PFS 4 m OS 10 m	OS higher in BRAF wt 12 vs 9 m
Kim et al. (73)	II randomized	Stage IV melanoma. No brain metastases.	1^st^ line	Carboplatin + Paclitaxel + Placebo (71 pts)	Carboplatin + Paclitaxel + Bevacizumab (143 pts)	PFS 4 vs 5 m	OS 9 vs 12 m OR 11/67 vs 36/141 DOR 8 vs 7 m G3-G5 toxicity 57 vs 45%	
Schuster et al. (74)	II	Stage IV melanoma. No brain metastases.	2^nd^ line	Bevacizumab (35 pts)	None	DCR 31%	PFS 2 m OS 9 m Toxicity 7/11 pts who had disease control developed hypertension	
Minor et al. (75)	II	Stage IV melanoma. No active brain metastases. cKit mutated.	2^nd^ line or further. No prior immunotherapy.	Sunitinib (10 pts)	None	ORR 3/4 in mutated cKIT pts; 1/6 in amplified or overexpressed cKIT pts.		
Mahalingam et al. (76)	II	Stage III unresectable or stage IV cutaneous melanoma. No active brain metastases.	2^nd^ or 3^rd^ line	Bevacizumab + Sorafenib (14 pts)	None	ORR 0% SD 21%	PFS 8 m G3-G4 toxicity 43%	Low VEGF values correlated with longer PFS
Ferrucci et al. (77)	II	Stage IV cutaneous melanoma. No brain metastases.	1^st^ line	Bevacizumab + Dacarbazine (40 pts)	None	ORR 19%	TTP 5 m Discontinuation 92% G3-G4 toxicity 22%	
NCT02158520	II randomized	Stage IV melanoma. No brain metastases	1^st^ line or further	Nab-Paclitaxel + Bevacizumab (12 pts)	Ipilimumab (12 pts)	PFS 129 vs 94 days	OS 18 vs 27 m ORR (2 vs 0 CR; 1 vs 1 PR) G3-G4 Toxicity (9 vs 7)	Recruitment completed. (24 enrolled pts vs 176 initially designed)

**Table 2:** Clinical trials on the association of antiangiogenic with immunotherapy or anti-BRAF/anti-MEK targeted therapy in advanced melanoma.

Reference	Phase	Clinical setting	Line of treatment in metastatic setting	Arm 1	Arm 2	Primary endpoints	Secondary endpoints	Further details
Hodi et al. (78)	I	Stage III unresectable or stage IV melanoma. No brain metastases	1^st^ or 2^nd^ line (46 pts)	Ipilimumab + Bevacizumab	None	ORR 8 PR, 22 SD	DCR 67% OS 25 m	
NCT01950390	II randomized	Stage III unresectable or IV cutaneous melanoma. No brain metastases	1^st^ or 2^nd^ line (168 pts)	Ipilimumab	Ipilimumab + Bevacizumab	OS	PFS ORR	Active, not recruiting. Results pending
Taylor et al. (79)	IB/II	Advanced solid tumors including melanoma	2^nd^ or 3^rd^ line (21 pts with melanoma)	Pembrolizumab + Lenvatinib	None	Safety	ORR at week 24 48% in melanoma (1 CR and 9 PR)	
Arance et al. (80)	II	Stage III unresectable or IV cutaneous melanoma.	2^nd^ or further line (103 pts)	Pembrolizumab + Lenvatinib	None	ORR 21.4%	PFS 4.2 m OS 13.9 m	
NCT03820986	III randomized	Stage III unresectable or IV cutaneous melanoma. No active brain metastases	1^st^ or 2^nd^ line (660 pts)	Pembrolizumab + Lenvatinib	Pembrolizumab + Placebo	PRS OS	ORR DOR Toxicity	Active, recruiting
NCT01495988	II	Stage IIIC unresectable or stage IV melanoma. BRAF V600E/V600K positive. No active brain metastases	1^st^ or further line (10 pts enrolled, vs initially designed 180 pts)	Vemurafenib + Cobimetinib + Bevacizumab (arm 2)	Vemurafenib + Cobimetinib (arm 1) Vemurafenib (arm 3) Vemurafenib + Bevacizumab (arm 4)	PFS	OS RR Toxicity (2 pts in arm 2. 1 of them, multi-organ failure)	Slow accrural, toxicity, change in priorities
NCT04356729	II	Stage III unresectable or IV cutaneous melanoma PDL1 negative. No active brain metastases	Any line (no prior immunotherapy)	Atezolizumab + Bevacizumab (30 estimated pts)	None	ORR	OS TTP DOR Safety Change in TILs	Not yet recruiting
NCT03175432	II	Stage IV melanoma with brain metastases. BRAF wt	Progression after anti-PD1	Atezolizumab + Bevacizumab + Cobimetinib	None	Intracranial ORR Safety	ORR DOR Neurocognitive function	Recruiting. Estimated 60 pts
NCT02681549	II	Stage IV melanoma or NSCLC with brain metastases	2^nd^ or further line	Pembrolizumab + Bevacizumab	None	Intracranial ORR	Need for steroids ORR PFS Safety Biomarkers	Recruiting. Estimated 53 pts
NCT03239145	I	Advanced solid tumors. No active brain metastases	2^nd^ or further line	Pembrolizumab + Trebananib	None	Maximum dose	ORR PFS OS	Recruiting. Estimated 60 pts

## Author Contributions

All authors listed have made a substantial, direct, and intellectual contribution to the work and approved it for publication.

## Conflict of Interest

The authors declare that the research was conducted in the absence of any commercial or financial relationships that could be construed as a potential conflict of interest.

The reviewer MM declared a shared affiliation with one of the authors, DQ, MG, to the handling editor at time of review.
